# Trimethylamine N-oxide (TMAO) for risk stratification after acute ischemic stroke: Results from the BIOSIGNAL cohort study

**DOI:** 10.1177/23969873251366192

**Published:** 2025-09-07

**Authors:** Johannes Frenger, Benjamin Jeker, Markus Arnold, Gerrit M Grosse, Thomas Pokorny, Laura P Westphal, Corinne Inauen, Giulio Bicciato, Marcel Arnold, Urs Fischer, Gian Marco De Marchis, Georg Kägi, Timo Kahles, Carlo W Cereda, Alejandro Bustamante, Joan Montaner, George Ntaios, Christian Foerch, Katharina Spanaus, Arnold von Eckardstein, Daniel Mueller, Mira Katan

**Affiliations:** 1 Department of Neurology & Stroke Center, University Hospital of Basel & University of Basel, Basel, Switzerland; 2 Department of Neurology, University Hospital Zurich, Zurich, Switzerland; 3 Department of Neurology, University Hospital and University of Bern, Bern, Switzerland; 4 Department of Neurology & Stroke Center, Cantonal Hospital St. Gallen, University Teaching and Research Hospital, St. Gallen, Switzerland; 5 Department of Neurology, Cantonal Hospital Aarau, Aarau, Switzerland; 6 Neurocentro della Svizzera Italiana, Stroke Center EOC, Lugano, Switzerland; 7 Stroke Unit, Department of Neurology, Hospital Universitari Germans Trias i Pujol, Germans Trias i Pujol Research Institute (IGTP), Barcelona, Spain; 8 Institute de Biomedicine of Seville, IBiS/Hospital Universitario Virgen del Rocío/CSIC/University of Seville, Seville, Spain; 9 Department of Neurology, Hospital Universitario Virgen Macarena, Seville, Spain; 10 Department of Internal Medicine, Faculty of Medicine, School of Health Sciences, University of Thessaly, Larissa, Greece; 11 Department of Neurology, RKH Hospital Ludwigsburg, Ludwigsburg, Germany; 12 Institute of Clinical Chemistry, University Hospital Zurich, Zurich, Switzerland; 13 Department of Clinical Chemistry, University Hospital of Basel & University of Basel, Basel, Switzerland; 14 Department of Chemistry, Solothurn Hospitals AG, Solothurn, Switzerland

**Keywords:** TMAO, trimethylamine-N-oxide, major adverse cardiovascular events, functional outcome, secondary prevention, risk stratification, ischemic stroke

## Abstract

**Introduction:**

Recent studies in stroke patients from predominantly Asian populations have underscored the significance of trimethylamine N-oxide (TMAO) as a valuable blood biomarker for predicting incident strokes and major adverse cardiovascular events (MACE). However, its prognostic role after ischemic stroke in other populations is not yet comprehensively investigated.

**Patients and methods:**

We measured plasma TMAO levels in 1726 acute ischemic stroke patients (within 24 h from symptom onset) from the multicenter BIOSIGNAL cohort. Using cox and logistic regression models adjusting for demographic and vascular risk factors, we investigated the association of TMAO with recurrent stroke, MACE within 365 days and functional outcome at 90 days after stroke.

**Results:**

TMAO levels were not associated with any risk of recurrent stroke (*n* = 108, unadj. HR per unit increase of log (TMAO) 1.15, 95% CI 0.88–1.51, adjust. HR 1.07, 95% CI 0.78–1.47) or MACE (*n* = 309, unadj. HR of log (TMAO) 1.10,95% CI 0.91–1.3, adjust. HR 0.90, 95% CI 0.74–1.09). There was an univariable positive association between higher TMAO plasma levels and unfavorable functional outcome, this association remained statistically significant in the multivariable analysis (unadj. OR of log (TMAO) 1.56, 95% CI 1.34–1.81, adjust. OR 1.28, 95% CI 1.04–1.57).

**Conclusion:**

In this large cohort of acute stroke patients from a predominantly White population, TMAO had no independent association with either recurrent stroke, or MACE or death. In univariable, and multivariable analysis, there was a significant association between TMAO and unfavorable functional outcome, which might not be clinically significant due to its low effect size. Therefore, TMAO seems not to be a clinically relevant biomarker for risk stratification after stroke.

## Graphical abstract

**Graphical abstract:**
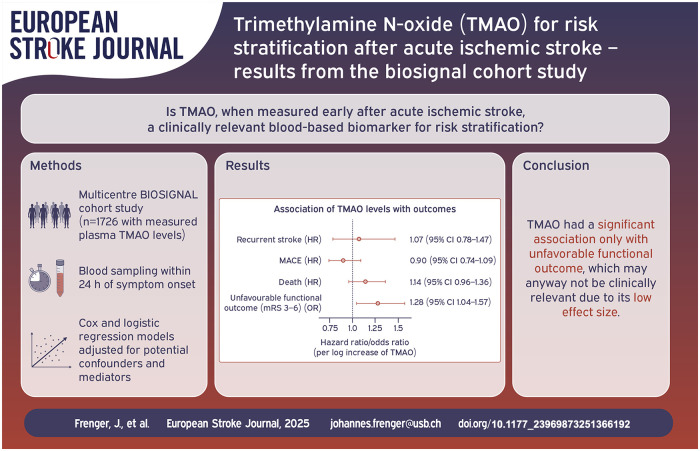

## Introduction

Ischemic Stroke is the second leading cause of death and disability with a yearly incidence of 13.7 million worldwide of which about 87% are ischemic strokes.^1^ Well defined risk factors such as hypertension, physical inactivity or diet account for a high percentage of stroke risk but do not fully explain all attributable stroke risk.^2^ Therefore, the identification of novel risk factors might help to open doors for new treatment strategies to lower both stroke incidence and recurrence. Being potentially modifiable due to its dietary origin, trimethylamine N-oxide (TMAO) emerged as a promising new biomarker in this regard.

TMAO is generated through the oxidation of trimethylamine (TMA) by hepatic flavin monooxygenases (FMOs).^3,4^ TMA in turn is synthesized by the gut microbiota using precursor molecules like betaine, choline or L-Carnitine.^5^ L-Carnitine and choline are very abundant in animal-derived nutrients like meat (especially red meat), eggs, milk and shellfish, while betaine is common in plants.^6,7^ Studies have highlighted different pathways how TMAO could contribute to atherosclerosis (AS) and thrombosis.^3,4^ Firstly, a promotion of AS via an autoimmunity axis was postulated, which is mediated by autoantigens that cause macrophages to endocytose lipids and thereby turn them into foam cells.^3^ Secondly, another link between TMAO and AS may exist via endovascular inflammation, which is a key factor in the early stages of atherosclerosis.^8,9^ TMAO has been shown to elevate proinflammatory cytokines such as interleukin-6, C-reactive protein, and reactive oxygen species in endothelial cells, while simultaneously downregulating nitric oxide production.^10^ And thirdly, besides promoting AS, TMAO might promote platelet hyperactivity and thereby increase the risk for thrombosis.^11,12^

Several studies have outlined a positive association between TMAO and cardiovascular diseases including incident stroke.^13,14^ For example, pooled data from two U.S. cohorts of 11,785 healthy individuals without a history of stroke demonstrated an association between TMAO and incident stroke in a multivariable model, after adjusting for demographic and vascular risk factors (HR for a doubling of TMAO: 1.11 [1.03–1.18]).^15^ The role of TMAO as a risk factor of recurrent stroke or poor stroke outcomes remains insufficiently defined. In a German cohort of 671 individuals experiencing their first-ever stroke, TMAO levels had a dose-dependent relationship with the risk of recurrent stroke as well as recurrent major adverse cardiovascular events (MACE).^16^ These findings were confirmed in a larger prospective study of 10,756 Chinese ischemic stroke patients.^17^ The associations of TMAO with recurrent stroke or MACE were closest in patients with small vessel disease. With regard to post-stroke recovery, an observational study involving 351 patients showed that elevated TMAO plasma levels measured during the acute phase of stroke predicted functional outcomes independently of other risk factors.^18^ However, another study of 196 patients with acute ischemic stroke^19^ found no significant association between TMAO levels and functional outcomes. A recent systematic review and meta-analysis, encompassing 40,061 stroke patients, unveiled a substantial correlation between TMAO and unfavorable outcomes after stroke, though the findings were dominated by 98% of data being derived from the Chinese cohort.^20^

The aim of this study is to analyze if TMAO levels measured in the acute phase after an ischemic stroke are associated with stroke recurrence, MACE, death, and functional outcome within 1 year after stroke in a well-characterized Caucasian population.^21^

## Methods

### Study design and patient cohort

The BIOSIGNAL (Biomarker Signature of Stroke Etiology, ClinicalTrial.gov NCT02274727) study is a prospective, observational, multicenter, inception cohort study to evaluate selected prognostic and etiological blood biomarkers in patients with confirmed acute ischemic stroke (AIS). A detailed description of the study was published elsewhere.^21^ Briefly, 1759 patients with AIS were enrolled between October 2014 and October 2017 at nine stroke centers in Europe. Patients with hemorrhagic stroke, transient ischemic attack (TIA) or patients discharged with a diagnosis other than ischemic stroke (i.e. stroke mimics) were excluded. Blood samples were obtained within 24 h of symptom onset. The BIOSIGNAL study was approved by all local ethics committees and conducted according to the principles expressed in the Declaration of Helsinki. All patients or their welfare guardians provided written informed consent. The identified data supporting the findings of this study are available from the corresponding author on reasonable request.

### Clinical data

Demographic variables, vital signs, vascular risk factors and stroke severity by the National Institute of Health Stroke Scale (NIHSS) were collected by stroke physicians. All participants underwent CT and/or MRI on admission. Participants received standard of care etiological workup containing 12-lead electrocardiography, >24 h continuous ECG-monitoring, transthoracic and/or transesophageal echocardiography, neurovascular ultrasound and/or CT or MR angiography. Stroke etiology was determined according to the TOAST-classification (trial of Org 10172 in Acute Stroke).^22^

Predefined outcomes were ischemic stroke recurrence, MACE, defined as recurrent cerebrovascular events, myocardial infarction, or cardiovascular death 1 year after stroke, and functional outcome 90 days after stroke.

Follow-up was performed either during an outpatient visit or with a structured telephone interview by trained stroke physicians. Assessment of functional outcome was performed using the modified Rankin Scale (mRS).^23^

### Biomarker measurement

Blood was drawn within 24 h of symptom onset during the first routine blood sampling in EDTA containing plastic tubes. Samples were immediately centrifuged at 3000*g* at 4°C for 20 min, aliquoted, and frozen at −80° C until the time of analysis. TMAO levels were assessed similarly as previously described and blinded to all clinical data.^24^ In brief, 400 μL of the internal standard TMAO-d9, dissolved in methanol, was added to the samples, which were then centrifuged at 11,700*g* for 10 min at 4°C. Fifty microliters of the supernatant were mixed with 50 μL of methanol for dilution. The sample was then analyzed using an Accucore HILIC column (50 × 2.1 mm, 2.6 μm particle size; Thermo Fisher Scientific, Reinach, Switzerland) with mobile phases set to pH 3. The following transitions were monitored using a QTrap 6500+ mass spectrometer (AB Sciex, Baden, Switzerland), operated in positive electrospray ionization mode: 76.1 → 59.1 (quantifier), 76.1 → 42.1 and 76.1 → 56.2 (qualifiers) for TMAO, and 85.1 → 68.1 for TMAO-d9.

### Statistical analysis

Discrete variables were expressed as counts (%) and continuous variables as medians and inter-quartile-ranges (IQR). Baseline demographics and risk factors were compared between groups using the chi square test, or Kruskal-Wallis test, as appropriate and indicated in the legends of figures or tables. To compare baseline characteristics and according to previous published research in this field, we stratified TMAO plasma levels into quartiles.

To determine the association of TMAO with outcome events (MACE, recurrent stroke, death), univariable (and multivariable) Cox proportional-hazards regression analyses were performed with TMAO divided into quartiles (according to previously published literature) and TMAO as continuous variable. The cox proportional hazard assumption was checked using a visual approach based on graphical diagnostics based on the scaled Schoenfeld residuals. Common logarithmic transformation (base 10) was performed for TMAO to transform to normality for skewed distribution. Hazard ratios (HR) and their 95% CI, with age, sex, hypertension, smoking, diabetes, renal function defined as estimated glomerular filtration rate > 60 mL/min /<60 mL/min (eGFR), stroke severity (divided into three groups: minor stroke (NIHSS 0–4), moderate stroke (NIHSS 5–8), major stroke (NIHSS 9–42), and cardioembolic stroke etiology covariates were estimated. We also reported the AIC and AUC values of the multivariable models, as well as the ΔAUC, which shows how much the AUC changed when TMAO was added to the multivariable model. Covariates were selected using a two-tracked approach. First, we used differences in the distribution of the baseline Table 1 to detect covariates that influence the outcome of interest or the distribution of TMAO. Second, a rational approach was used to select covariates described in previously published literature that have a known impact on TMAO levels and/or the outcome of interest. Cumulative incidence curves were plotted using TMAO quartiles, and estimates were compared with the log-rank test. To assess the association of TMAO levels with unfavorable outcome (defined as mRS 3–6 at 90 days) uni- and multivariable logistic regression models were built with the same predefined covariates but with addition of pre-stroke mRS status (dichotomous variable; 0–2 favorable, 3–6 unfavorable), such as a revascularization therapies (Thrombolysis and/or Thrombectommy) as dichotomous variable. . For prediction of clinical events, patients that were lost to follow up were censored at the time of loss-to follow up (however only six patients (0.3%) were lost to follow up). For analysis of MACE, patients who died a non-cardiovascular death were censored at the time of death.

**Table 1.** Baseline characteristics with TMAO divided into quartiles.

TMAO plasma level	Quartile 1 (*N* = 432)	Quartile 2 (*N* = 431)	Quartile 3 (*N* = 431)	Quartile 4 (*N* = 432)	*p*-Value
Quartile range (μmol/L)	0.65–2.78	2.79–4.01	4.02–6.13	6.14–164.82	
*Demographics*					
Age (median [IQR])	67 [54, 78]	73 [61, 80]	76 [68, 82]	80 [72, 86]	<0.001
Sex, male (%)	241 (55.8)	274 (63.6)	257 (59.6)	234 (54.2)	0.024
Premorbid mRS (favorable) (%)	400 (92.8)	401 (93.3)	392 (91%)	372 (86%)	<0.001
*Vital signs*					
RR sys (median [IQR])	150 [134, 166]	156 [140, 174]	155 [140, 172]	158 [140–175]	<0.001
Heart rate (median [IQR])	80 [69, 90]	80 [68, 88]	76 [68,89]	77 [67, 88]	0.372
Temperature (median [IQR])	36.6 [36.2, 36.9]	36.6 [36.2,36.8]	36.6 [36.3,36.9]	36.6 [36.2,36.9]	0.550
*Medical history*					
Hypertension (%)	260 (60.2)	305 (70.8)	323 (74.9)	368 (85.2)	<0.001
Hyperlipidemia (%)	278 (64.4)	301 (69.8)	297 (68.9)	305 (70.6)	0.194
Smoking (%)	113 (26.6)	108 (25.2)	92 (21.4)	70 (16.4)	0.002
Alcohol abuse (%)	23 (5.5)	27 (6.3)	31 (7.4)	17 (4.1)	0.207
History of stroke/TIA (%)	68 (15.7)	51 (11.8)	73 (16.9)	103 (23.8)	<0.001
Positive CVD family history (%)	66 (16.3)	63 (15.5)	50 (12.5)	34 (8.9)	0.009
Dementia (%)	21 (4.9)	20 (4.6)	26 (6.1)	40 (9.3)	0.017
Diabetes (%)	59 (13.7)	50 (11.6)	72 (16.7)	110 (25.5)	<0.001
*Laboratory measurements*					
Hb (median [IQR] g/L)	141 [130, 151]	142 [131, 151]	138 [125, 147.75]	133 [118, 144]	<0.001
Leukocytes (median [IQR] g/L)	8 [7, 11]	8 [7, 1]	8 [7, 1]	8 [6, 1]	0.730
Thrombocytes (median [IQR| g/L)	226.5 [188, 274]	227 [190, 268]	224 [183, 269]	216 [181, 265]	0.107
GFR >60 mL/min (%)	329 (77.6)	240 (56.6)	199 (46.5)	110 (25.9)	<0.001
Cholesterol (median [IQR] mmol/L)	4.9 [4.1, 5.6]	5 [4.23, 5.8]	4.9 [4.2, 5.6]	4.5 [3.7, 5.4]	<0.001
Cholesterol LDL (median [IQR] mmol/L)	2.7 [2.05, 3.4]	2.87 [2.1, 3.6]	2.8 [2.1, 3.48]	2.5 [1.7, 3.2]	<0.001
Cholesterol HDL (median [IQR] mmol/L)	1.36 [1.08, 1.77]	1.31 [1.08, 1.6]	1.35 [1.1, 1.64]	1.2 [1, 1.5]	<0.001
Triglycerides (median [IQR] mmol/L)	1.11 [0.78, 1.63]	1.23 [0.9, 1.86]	1.31 [0.96, 1.9]	1.41 [1.04, 2.04]	0.107
Glucose (median [IQR] mmol/L)	6.40 [5.70, 7.40]	6.40 [5.60, 7.47]	6.20 [5.60, 7.60]	6.40 [5.60, 7.80]	0.309
*Information index event*					
Stroke severity - minor stroke (NIHSS 0-4)	187 (43.5)	204 (47.4)	213 (49.4)	187 (43.4)	0.009
- Moderate stroke (NIHSS 5-8)	78 (18.1)	93 (21.6)	103 (23.9)	87 (20.2)	
- Major stroke (NIHSS 9-42)	165 (38.4)	133 (30.9)	115 (26.7)	157 (36.4)	
Etiology – TOAST LAA (%)	67 (15.5)	61 (14.2)	59 (13.7)	64 (14.8)	0.882
Etiology – TOAST CE (%)	133 (30.8)	124 (28.8)	151 (35.0)	162 (37.6)	0.027
Etiology – TOAST SVD (%)	40 (9.3)	46 (10.7)	47 (10.9)	47 (10.9)	0.833
Etiology – TOAST OTH (%)	41 (9.5)	23 (5.3)	14 (3.2)	12 (2.8)	<0.001
Etiology – TOAST UNK (%)	151 (35.0)	177 (41.1)	160 (37.1)	146 (33.9)	0.132
Intravenous thrombolysis (%)	190 (43.9)	172 (39.9)	175 (40.6)	160 (37.0)	0.223
Mechanical recanalization (%)	106 (24.8)	89 (20.7)	70 (16.3)	64 (14.8)	<0.001

mRS: modified Rankin Scale; RR sys: first measured systolic blood pressure; TIA: transient ischemic attack; HB: hemoglobin; GFR: glomerular filtration rate; LAA: large artery atherosclerosis; CE: cardioembolic stroke; SVD: small vessel disease; OTH: stroke of other determined cause; UNK: stroke of undetermined cause.

To assess risk differences in different stroke etiology subgroups, the data were stratified into five subgroups based on the underlying stroke etiology of the index stroke, as determined by the TOAST classification. Univariate and multivariate Cox proportional-hazards regression analyses were conducted to assess recurrent stroke, major adverse cardiovascular events (MACE), and death within these subgroups. Due to limited event rates multivariable analysis was only performed with age and eGFR and only if the event rate was above 30. For assessment of functional outcome risk uni- and multivariable (age and eGFR) logistic regression models with subgroups were built. *p*-Values < 0.05 were considered statistically significant. To account for multiple comparisons, *p*-values were adjusted using the Holm-Bonferroni method. Statistical analysis was performed, and tables and figures were designed with R 4.3.1.

## Results

### Baseline characteristics

**Figure 1.**
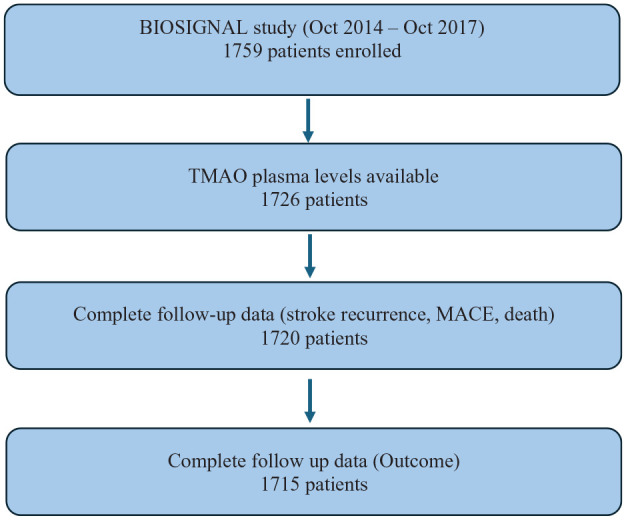
BIOSIGNAL study flowchart. MACE: major adverse cardiovascular event.

A total of 1759 participants with an AIS were consecutively enrolled in the study and TMAO plasma levels were available for 1726 participants (see Figure 1). Plasma levels of 33 participants could not be quantified due to insufficient sample volume. Of the 1726 participants, 1678 (97%) self-identified as White, 8 as Asian, 6 as Latino/a, 3 as Black, 7 as Other/Multiracial, and 24 were unable to specify their ethnicity. Mean TMAO value was 5.79 μmol/L (SD 8.14), median TMAO value was 4.01 μmol/L (IQR 3.35). TMAO levels were stratified in quartiles (first quartile (Q1): 0.65–2.78 μmol/L, second quartile (Q2): 2.79–4.01 μmol/L, third quartile (Q3): 4.02–6.13 μmol/L and fourth quartile (Q4): 6.14–164.82 μmol/L).

Baseline characteristics stratified by TMAO quartiles are displayed in Table 1. Compared to Q1, patients in Q4 were older (*p* ⩽ 0.001), less often male (*p* = 0.024), had a higher premorbid mRS (*p* < 0.001), had a higher burden of hypertension (*p* ⩽ 0.001), diabetes (*p* ⩽ 0.001), were less often smokers (*p* = 0.002) and had less prevalence of a positive family history regarding cerebrovascular disease (*p* = 0.009). Also, history of stroke/TIA (*p* ⩽ 0.001) or dementia (*p* = 0.017) was more frequent in Q4 compared to Q1. Normal renal function defined as eGFR > 60 mL/min was rarer in patients with higher TMAO levels (*p* < 0.001). Furthermore, High-density lipoprotein cholesterol (HDL-C), Low-density lipoprotein cholesterol (LDL) and Cholesterol were lower in Q4 than in Q1 (*p* ⩽ 0.001). No significant differences between TMAO quartiles were found for prevalence of hyperlipidemia, history of alcohol abuse, leukocyte count, and thrombocyte count. Regarding the index event, patients in Q1 and Q4 tended to have higher NIHSS scores compared to those in Q2 and Q3 (*p* = 0.013). Furthermore, patients in Q4 more frequently had cardioembolic strokes (*p* = 0.027) and less frequently had strokes of other determined causes (*p* < 0.001). No significant differences were observed between TMAO levels and other stroke etiologies.

### Stroke recurrence

**Figure 2.**
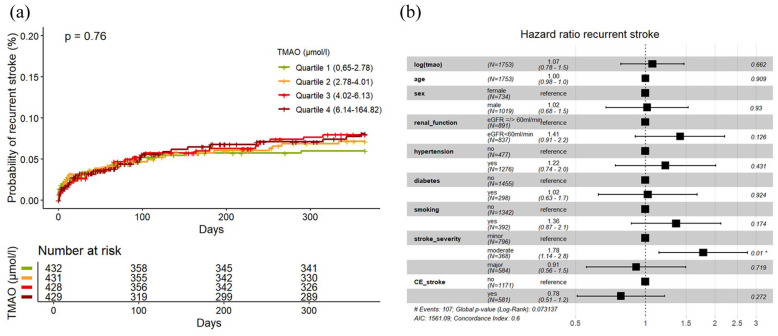
(a) (Left): Cumulative incidence curves of recurrent strokes according to quartiles of circulating TMAO level, *p*-value for log-rank test. (b) (Right): Forest Plot of HR for recurrent strokes in multivariable Cox-Model with covariate effect size. CE_stroke: cardioembolic stroke according to TOAST-classification.

Out of 1726 participants, 1720 had complete follow-up with information about stroke recurrence, MACE and death. During the 1 year of follow-up, we observed a total of 112 patients (6%) with recurrent stroke. As shown in the cumulative incidence curve (Figure 2(a)), there was no association between TMAO quartiles with stroke recurrence (univariable Q4 vs Q1 HR 1.30, 95% CI 0.75–2.23). The same was observed in univariable and multivariable Cox-regression models with log (TMAO) (HR (log increase) 1.15, 95% CI 0.88–1.51, and 1.07, 95% CI 0.78–1.47, AIC 1561.09, AUC 0.621, ΔAUC 0.02, respectively; Figure 2(b)). After stratifying the data into subgroups based on the underlying stroke etiology, no specific etiology was identified in which TMAO was associated with stroke recurrence in both univariate and multivariate analyses (see Supplemental Material).

### Occurrence of MACE

**Figure 3.**
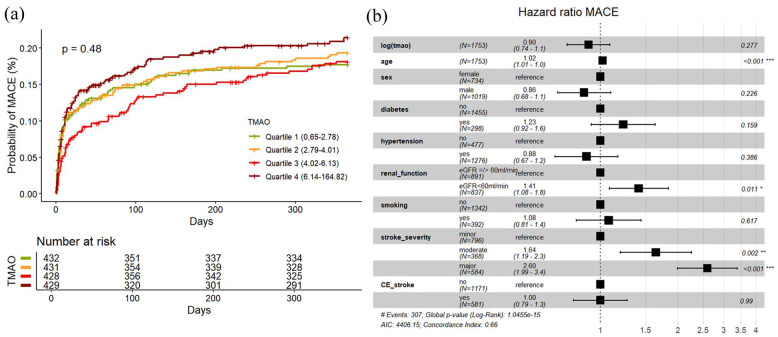
(a) (Left): Cumulative incidence curves of MACE occurrence according to quartiles of circulating TMAO level with log-rank test *p*-value. (b) (Right): Forest Plot of HR for MACE in multivariable Cox-Model with shown covariate effect size. CE_stroke: cardioembolic stroke according to TOAST-classification.

MACE occurred in 320 participants (19%) within 1 year after index event. As shown in Figure 3(a), cumulative event estimates for MACE did not differ significantly between predefined TMAO quartiles (univariable cox regression Q4 vs Q1 HR 1.22, 95% CI 0.90–1.66,). Neither univariable nor multivariable Cox-model analyses showed any association of log (TMAO) with MACE (HR (log increase) 1.1, 95% CI 0.91–1.3) and HR 0.90, 95% CI 0.74–1.09, AIC 4406.15, AUC 0.663, ΔAUC +0.00 respectively, Figure 3(b)). After stratifying the data into subgroups based on the underlying stroke etiology, no specific etiology was identified in which TMAO was associated with MACE in both univariate and multivariate analyses (Supplemental Material).

### Occurrence of all cause death

**Figure 4.**
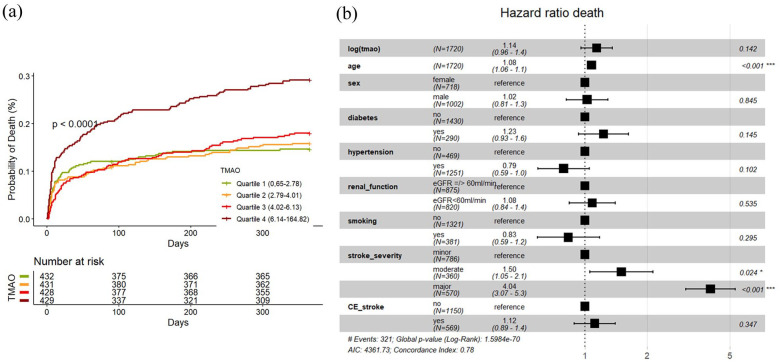
(a) (Left): Cumulative incidence curves of mortality according to quartiles of TMAO with log-rank test *p*-value. (b) (Right): Forest Plot of HR for mortality in multivariable Cox-Model with shown covariates. CE_stroke: cardioembolic stroke according to TOAST-classification.

A total of 333 deaths (19%) were observed within 1 year after index event. As presented in Figure 4(a), cumulative event estimates showed increasing mortality with increasing TMAO quartiles (univariable cox regression Q4 vs Q1 HR 2.13, 95% CI 1.58–2.89). Also, univariable Cox-models showed increased hazards for mortality for increased log (TMAO) levels (HR (log increase) 1.48, 95% CI 1.29–1.70). In the multivariable analysis there was no association between log (TMAO) and death (HR 1.14 (0.96–1.36), AIC 4361.73, AUC 0.806, ΔAUC 0.01, Figure 4(b)). After stratifying the data into subgroups based on the underlying stroke etiology, there was an univariable significant association between TMAO and death in the cardioembolic subgroup (HR 1.62, 95% CI 1.29–2.04, adjusted *p*-value < 0.001), which disappeared in the multivariable analysis (HR 1.15, 95% CI 0.89–1.51, adjusted *p*-value 1.0) (Supplemental Material).

### TMAO and prediction of stroke outcome

**Figure 5.**
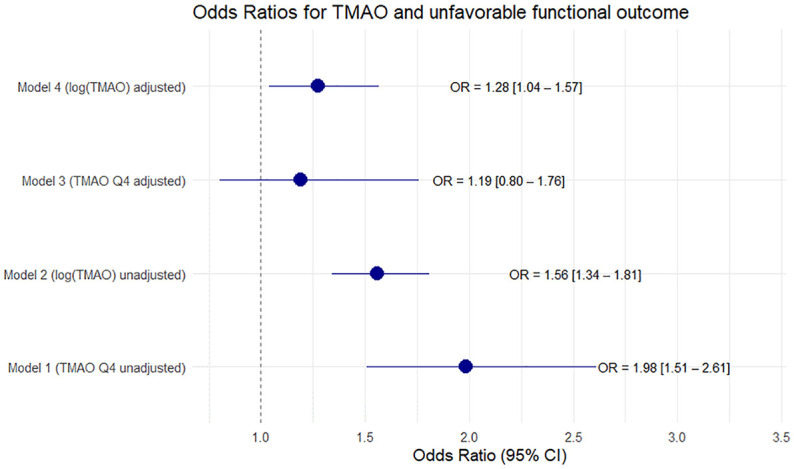
Forest plot with Odds Ratios for unfavorable outcome after 90 days in uni- and multivariable (adjusted for age, sex, diabetes, hypertension, renal function, smoking, premorbid mRS, stroke-severity, cardioembolic stroke (TOAST 2) and revascularization therapies) logistic regression with log (TMAO) and TMAO quartiles.

Out of 1726 participants, MRS data at 90 days were available for 1715 patients. 1048 (61%) had an outcome of mRS 1 or 2 and 667 (39%) had an outcome of mRS ⩾ 3. Upon logistic regression analysis of TMAO quartiles higher quartiles are associated with unfavorable outcome with a risk increase of 98% for unfavorable outcome when comparing Q1 with Q4 in the univariable model (OR Q4 vs Q1:1.98, 95% CI 1.51–2.62). However, no significant effect could be shown after adjustment with predefined covariates (OR Q4 vs Q1:1.33, 95% CI 0.88–2.00, AIC 1514.18, AUC 0.86). A similar finding is shown in Figure 5. In the univariable binary logistic regression analysis log (TMAO) was correlated significantly with bad outcome (OR (log increase) 1.56, 95% CI 1.34–1.81).

After adjustment for predefined covariates, the correlation remained statistically significant (OR (log increase) 1.28, 95% CI 1.04–1.57, AIC 1565.04, AUC 0.851, ΔAUC 0.02 ). After stratifying the data by underlying stroke etiology, a significant association between TMAO and adverse functional outcomes was found in the univariate analysis for the cardioembolic stroke and small vessel disease subgroups (cardioembolic stroke: OR (log increase) 1.69, 95% CI 1.31–2.21, adjusted *p*-value 0.0007; small vessel disease: OR 3.01, 95% CI 1.68–5.87, adjusted *p-*value 0.0000). However, no significant association was observed in the multivariate analysis (see Supplemental Material).

## Discussion

In the to date largest prospectively investigated predominantly White cohort of patients with acute ischemic stroke we did not find any significant independent association of TMAO plasma levels with recurrent stroke, MACE, mortality and only a weak association with unfavorable functional outcome.

The levels of measured TMAO and their distribution were comparable to values reported in previously published Asian and European studies.^16,19,25^ The number of outcome events (6% recurrent strokes, 18% MACE, 19% death, and 39% unfavorable outcome) is comparable to those reported in other large studies in the literature, which is why we consider our dataset to be reliable for a typical stroke cohort.^26–28^ Our findings contradict those of a study of 671 German patients with acute ischemic stroke,^16^ which reported significant associations of TMAO plasma levels with recurrence of stroke as well as MACE. However, while the association of TMAO with MACE was reported to persist after multivariate adjusting for cardiovascular risk factors, no data were shown on the effect of multivariate adjustments on the prognostic value of TMAO toward recurrent stroke. The timing of the blood sampling was not defined, making it difficult to compare the results with those of our study. As described recently,^19^ the timing of blood-sampling has substantial impact on the TMAO plasma levels after stroke. Based on these results, a standardized blood sampling either within 24 h after admission or 3 months thereafter was recommended. A possible explanation for altered levels after stroke is the severe gut dysbiosis that occurs after large infarctions.^29^ In a Chinese cohort of 10,756 patients with acute stroke or TIA, high plasma levels of TMAO or choline increased the risk of recurrent strokes after 1 year, especially in the subgroup of patients with small vessel disease with an adjusted HR of 1.43 (1.03–2.00).^17^ In that study, blood was sampled within 24 h after onset of stroke and under fasting conditions. However, exclusively Chinese patients were included in this study and the authors recommended verification of the results in other ethnicities. To our knowledge, the results of the Asian cohort study was not adjusted for multiple testing, which is a notable limitation. Additionally, the reported effect in the small vessel disease subgroup ((*n* = 2237, HR 1.43, [95% CI, 1.03-2.00]) appears to be very small, which is why we consider it negligible.

The association of TMAO with survival after acute ischemic stroke was previously investigated in two Chinese studies with 351 and 225 patients.^18,30^ Both studies showed a positive association of elevated TMAO levels with mortality in both univariable und multivariable testing. The study with 225 patients was not adjusted for eGFR, which is a notable limitation. We did not find any data on stroke patients regarding all-cause mortality in a white population and therefore, a direct comparison of our results with other studies is not possible.

Several hypotheses have been proposed regarding the mechanisms by which TMAO may negatively influence functional outcomes, including the activation of inflammatory pathways that disrupt the blood-brain barrier and increase platelet reactivity. A recently published study demonstrated that fecal microbiota transplantation from human donors with low or high TMAO production into germ-free mice indicates that both TMAO generation and stroke severity are transmissible traits.^31^ However, the evidence from observational studies showing a clear association between TMAO levels and functional outcomes after stroke remains a subject of ongoing debate. With respect to functional outcome, our findings are in line with the results of a systematic review and meta-analysis including 40,061 stroke patients,^20^ in which the risk of poor functional outcome after stroke with increasing TMAO levels (OR 1.58 (95% CI 1.26–1.99). In this meta-analysis 13 of 15 studies were conducted in China. Interestingly, none of the two other studies, which were performed in Germany^19,32^ found any significant association between TMAO and outcome. Therefore, our study is the first to demonstrate a statistically significant association between TMAO levels and unfavorable outcome in a European population. However, the effect size (OR 1.28 per log increase) appears modest with only a minimal increase of AUC 0.02 when adding TMAO to the multivariable model. Therefore, TMAO does not seem to be a clinically relevant novel prognostic marker to guide clinical decision making.

Our results for recurrent stroke, MACE and death contrast with those derived from Asian populations. This raises the question of whether significant differences exist between different ethnic groups. This question was addressed by the analysis of pooled data from two US-cohorts of 11,785 healthy individuals without prior stroke history as mentioned previously^15^ about 7% of whom were Asian-American. Within a median follow-up time of 15 years TMAO had a closer association with risk of incident stroke among Asian-American individuals (HR 1.57, 95% CI 0.84–2.94) than among other ethnic groups, for example White individuals (HR 1.09, 95% CI 1.00–1.18)). Due to the small group size the difference between ethnicities was not significant. Also, in another study of 1232 patients on hemodialysis the risk association between TMAO plasma concentration and cardiovascular events varied by race. The differences may result from differences in diet and microbiome between ethnic groups.^33^

In our study, the association of TMAO with mortality, became substantially weaker and statistically insignificant by adjustment for age and renal function. As shown in Table 1 and reported by others, TMAO increases substantially both with aging and decreasing eGFR.^34,35^ Also, others found that associations of TMAO with cardiovascular endpoints were not independent of renal function.^36^ This probably reflects the fact that about 95% of TMAO is eliminated by the kidney.^37^ The risk of chronic kidney disease (CKD) is even increasing with higher TMAO levels^38^ either reflecting a causal role of TMAO for CKD or indicating a very sensitive response of TMAO to declining kidney function. In any case elevated TMAO plasma concentrations may be a bystander of impaired renal function which is a well-known risk factor of stroke and premature death. Furthermore, elevated TMAO levels are associated with other cardiovascular risk factors such as diabetes, hypertension, and lipid levels, which might confound the associations of TMAO with clinical endpoints. Our results suggest that TMAO is more likely a marker of various cardiovascular risk factors that worsen mortality after ischemic stroke, rather than a direct cause of mortality.

This study has some limitations. First, we have no information on dietary habits, neither on long nor on short term. The latter would be of special interest as TMAO levels increase rather quickly after consumption of carnitine- or choline-rich meals such as meat or eggs,^3,4^ while the chronic effect of diet on TMAO levels is rather small.^8,39^ Therefore, we cannot exclude that dietary habits or fasting before blood sampling affect TMAO plasma concentrations. Second, plasma TMAO values were only measured at a single time point within the acute post-stroke period, and we did not measure the plasma level of TMAO during follow-up or at additional time-points. This is an interesting research question because of the generally high intraindividual variability of TMAO^40^ and because of the development of gut dysbiosis after stroke.^19,29^ Although of less clinical value, it still remains unknown whether TMAO might play a role for outcome prediction in the longer course after an acute event and if TMAO would be suited to assess the compliance to secondary preventive measures. Third, we have no information about patients gut microbiota composition. This could be of interest with respect to differences between ethnic groups.

Despite its limitations, our study has several strengths. First, it features a large sample size: to our knowledge, the BIOSIGNAL cohort is the largest White cohort of stroke patients with measured TMAO plasma samples to date. Second, the cohort is well-characterized and has a high percentage of complete data, allowing us to carefully adjust for several risk factors reducing the impact of residual confounding. Third, blood sampling occurred early, within 24 h of admission. This is particularly important, as previous studies have shown that TMAO levels are typically elevated during the acute phase and decrease after 48 h. While the optimal timing for measuring TMAO after a stroke remains a topic of debate, experts recommend measurement within 24 h of the event. This recommendation was adhered to in the BIOSIGNAL study.^19^ Fourth, our statistical approach using log (TMAO) as a continuous variable rather than dividing the data into quartiles, as many previous studies have done. This approach prevents the loss of relevant information that can occur with data transformation.

## Conclusion

Based on our findings from the largest predominantly White stroke cohort studied to date, we could not confirm TMAO as an independent biomarker for stroke recurrence, MACE and all-cause mortality, but found association toward unfavorable functional outcome, which might not be clinically significant due to its low effect size. Our results suggest that TMAO is more likely a marker of various cardiovascular risk factors that worsen mortality after ischemic stroke, rather than a direct cause of mortality.

## Supplemental Material

sj-docx-1-eso-10.1177_23969873251366192 – Supplemental material for Trimethylamine N-oxide (TMAO) for risk stratification after acute ischemic stroke: Results from the BIOSIGNAL cohort study Supplemental material, sj-docx-1-eso-10.1177_23969873251366192 for Trimethylamine N-oxide (TMAO) for risk stratification after acute ischemic stroke: Results from the BIOSIGNAL cohort study by Johannes Frenger, Markus Arnold, Gerrit M Grosse, Thomas Pokorny, Laura P Westphal, Corinne Inauen, Giulio Bicciato, Marcel Arnold, Urs Fischer, Gian Marco De Marchis, Georg Kägi, Timo Kahles, Carlo W Cereda, Alejandro Bustamante, Joan Montaner, George Ntaios, Christian Foerch, Katharina Spanaus, Arnold von Eckardstein, Daniel Mueller and Mira Katan in European Stroke Journal
