# Supplementary material for: Trimethylamine N-oxide (TMAO) for risk stratification after acute ischemic stroke: Results from the BIOSIGNAL cohort study
Source: Eur Stroke J. 2025 Sep 7:23969873251366192. Online ahead of print. doi: 10.1177/23969873251366192 (PMC12417456; doi:10.1177/23969873251366192)
Supplement: sj-docx-1-eso-10.1177_23969873251366192 – Supplemental material for Trimethylamine N-oxide (TMAO) for risk stratification after acute ischemic stroke: Results from the BIOSIGNAL cohort study [file sj-docx-1-eso-10.1177_23969873251366192.docx]

**Supplemental material**

| **TOAST Subtype** | **n** | **Recurrent stroke events** | **Hazard Ratios (95% CI) with p-value and adjusted p-value (Holm-Bonferroni-Method)** | | |
| --- | --- | --- | --- | --- | --- |
|  |  |  | HR | p-value | Adjusted p-value |
| LAA | 251 | 23 |  |  |  |
| - log(TMAO) univariable |  |  | 0.84 (0.43-1.65) | 0.605 | 0.999 |
| - log(TMAO) multivariable* |  |  | 1.37 (0.34-1.54) | 0.403 | 0.999 |
| CE | 568 | 31 |  |  |  |
| - log(TMAO) univariable |  |  | 1.59(0.96-2.62) | 0.070 | 0.559 |
| - log(TMAO) multivariable* |  |  | 1.26(0.71-2.25) | 0.426 | 0.999 |
| SVD | 179 | 12 |  |  |  |
| - log(TMAO) univariable |  |  | 1.53 (0.80-2.94) | 0.197 | 0.999 |
| Others | 88 | 7 |  |  |  |
| - log(TMAO) univariable |  |  | 1.24(0.53-2.87) | 0.620 | 0.999 |
| Unknown | 633 | 39 |  |  |  |
| - log(TMAO) univariable |  |  | 0.94(0.57-1.55) | 0.890 | 0.999 |
| - log(TMAO) multivariable* |  |  | 0.84(0.48-1.48) | 0.556 | 0.999 |

Table 2: TMAO and stroke recurrence stratified by stroke subtype (multivariable* adjusted for age and renal function) LAA = large artery atherosclerosis, CE = cardioembolic stroke, SVD = small vessel disease, OTH = stroke of other determined cause, UNK = stroke of undetermined cause)

| **TOAST Subtype** | **n** | **MACE events** | **Hazard Ratios (95% CI) with p-value and adjusted p-value (Holm-Bonferroni-Method)** | | |
| --- | --- | --- | --- | --- | --- |
|  |  |  | HR | p-value | Adjusted p-value |
| LAA | 251 | 48 |  |  |  |
| log(TMAO) univariable |  |  | 1.12 (0.74-1.74) | 0.890 | 0.999 |
| log(TMAO) multivariable* |  |  | 0.88 (0.54-1.45) | 0.636 | 0.999 |
| CE | 568 | 114 |  |  |  |
| log(TMAO) univariable |  |  | 1.12(0.85-1.49) | 0.420 | 0.999 |
| log(TMAO) multivariable* |  |  | 0.81(0.59-1.12) | 0.208 | 0.999. |
| SVD | 179 | 18 |  |  |  |
| log(TMAO) univariable |  |  | 1.49(0.86-2.56) | 0.152 | 0.999 |
| Others | 88 | 19 |  |  |  |
| log(TMAO) univariable |  |  | 0.91(0.47-1.74) | 0.770 | 0.999 |
| Unknown | 633 | 121 |  |  |  |
| log(TMAO) univariable |  |  | 1.00(0.76-1.31) | 0.978 | 0.999 |
| log(TMAO) multivariable* |  |  | 0.8(0.59-1.11) | 0.192 | 0.999 |

Table 3: TMAO and MACE stratified by stroke subtype (multivariable* adjusted for age and renal function). LAA = large artery atherosclerosis, CE = cardioembolic stroke, SVD = small vessel disease, OTH = stroke of other determined cause, UNK = stroke of undetermined cause)

| **TOAST Subtype** | **n** | **Deaths** | **Hazard Ratios (95% CI) with p-value and adjusted p-value (Holm-Bonferroni-Method)** | | |
| --- | --- | --- | --- | --- | --- |
|  |  |  | HR | p-value | Adjusted p-value |
| LAA | 251 | 35 |  |  |  |
| - log(TMAO) univariable |  |  | 1.72 (1.11-2.67) | 0.016 | 0.109 |
| - log TMAO multivariable* |  |  | 1.147 (0.65-2.01) | 0.633 | 0.999 |
| CE | 568 | 149 |  |  |  |
| - log(TMAO) univariable |  |  | 1.62(1.29-2.04) | <0.001 | <0.001 |
| - log(TMAO) multivariable* |  |  | 1.15(0.89-1.51) | 0.295 | 0.999 |
| SVD | 179 | 15 |  |  |  |
| - log(TMAO) univariable |  |  | 1.30(0.67-2.51) | 0.441 | 0.999 |
| Others | 88 | 12 |  |  |  |
| - log(TMAO) univariable |  |  | 1.25(0.66-2.38) | 0.49 | 0.999 |
| Unknown | 633 | 121 |  |  |  |
| - log(TMAO) univariable |  |  | 1.31(1.03-1.68) | 0.0295 | 0.177 |
| - log(TMAO) multivariable* |  |  | 1.0(0.75-1.32) | 0.981 | 0.999 |

Table 4 : TMAO and death stratified by stroke subtype (multivariable* adjusted for age and renal function). LAA = large artery atherosclerosis, CE = cardioembolic stroke, SVD = small vessel disease, OTH = stroke of other determined cause, UNK = stroke of undetermined cause)

| **TOAST Subtype** | **n** | **Unvafourable outcomes (mrs 3-6)** | **Odds Ratios (95% CI) with p-value and adjusted p-value (Holm-Bonferroni-Method)** | | |
| --- | --- | --- | --- | --- | --- |
|  |  |  | OR | p-value | Adjusted p-value |
| LAA | 249 | 97 |  |  |  |
| log(TMAO) univariable |  |  | 1.57(1.05-2.37) | 0.029 | 0.222 |
| log TMAO multivariable* |  |  | 1.29(0.83-2.03) | 0.251 | 0.999 |
| CE | 568 | 259 |  |  |  |
| log(TMAO) univariable |  |  | 1.69(1.31-2.21) | <0.001 | <0.001 |
| log(TMAO) multivariable* |  |  | 1.06(0.78-1.46) | 0.689 | 0.999 |
| SVD | 177 | 46 |  |  |  |
| log(TMAO) univariable |  |  | 3.01(1.68-5.87) | <0.001 | <0.001 |
| log(TMAO) multivariable* |  |  | 2.05(1.12-4.10) | 0.028 | 0.222 |
| Others | 88 | 34 |  |  |  |
| log(TMAO) univariable |  |  | 1.47(0.81-2.88) | 0.033 | 0.222 |
| log(TMAO) multivariable* |  |  | 1.20(0.61-2.35) | 0.579 | 0.999 |
| Unknown | 632 | 230 |  |  |  |
| log(TMAO) univariable |  |  | 1.27(0.99-1.62) | 0.053 | 0.266 |
| log(TMAO) multivariable* |  |  | 0.98(0.75-1.29) | 0.907 | 0.999 |

Table 5 : Table 5: TMAO and outcome stratified by stroke subtype (multivariable* adjusted for age and renal function). LAA = large artery atherosclerosis, CE = cardioembolic stroke, SVD = small vessel disease, OTH = stroke of other determined cause, UNK = stroke of undetermined cause)
